# Editorial Comment: Diagnostic Assessment of Lower Urinary Tract Symptoms in Men Considering Prostate Surgery: A Noninferiority Randomised Controlled Trial of Urodynamics in 26 Hospitals

**DOI:** 10.1590/S1677-5538.IBJU.2021.05.03

**Published:** 2021-07-23

**Authors:** Jorge Moreno-Palacios

**Affiliations:** 1 IMSS UMAE Hospital de Especialidades CMN Servicio Urología Ciudad de México México Servicio Urología. UMAE Hospital de Especialidades CMN, Siglo XXI, IMSS, Ciudad de México

Marcus J Drake^1^, Amanda L Lewis^2^, Grace J Young^2^, Paul Abrams^3^, Peter S Blair^2^, Christopher Chapple^4^, et al.

Eur Urol 2020 Nov;78(5):701-710.

## COMMENT

Clinical evaluation of lower urinary tract symptoms (LUTS) in males includes: medical history, symptom score questionnaires, frequency charts and bladder diaries, physical examination, urinalysis, prostatic specific antigen, and in some cases assessment of the renal function, postvoid residual measurement and uroflowmetry. There has been a debate about the use of pressure flow studies (PFS) in this population, the major goal of urodynamics is to explore the functional mechanisms of LUTS, to identify risk factors for adverse outcomes and to provide information for shared decision-making. However, the guidelines recommendations are for PFS only in individual patients for specific indications prior to invasive treatment or when evaluation of the underlying pathophysiology of LUTS is warranted. (With a weak Strength rating) (1). The ideal information to answer this question should came from a randomized controlled trial (RCT) in which the expected outcomes could be surgical results, change the offered treatment and cost benefit comparing regular clinical work up versus UDS (in theory with better outcomes due to a more detailed information).

UPSTREAM is, noninferiority, randomised controlled trial in men with bothersome LUTS, in whom surgery was an option. The primary outcome was the International Prostate Symptom Score (IPSS) 18 mo after randomisation, with a noninferiority margin of 1 point. Urological surgery rates were a key secondary outcome. 427 and 393 patients were assigned to the UDS and routine care group respectively. For the primary outcome, the UDS arm demonstrated noninferiority for patient-reported LUTS, compared with routine care at 18 mo, with a difference in the mean IPSS of −0.33. The hypothesised reduction in surgery rates in the UDS arm was not shown at 18 mo. The results reported were: 38% (153/408) in the UDS arm received surgery during the 18-mo period, compared with 36% (138/384) in the routine care arm (odds ratio [OR] 1.05 [95% CI 0.77,1.43] which conclude that routine use of UDS in the evaluation of uncomplicated LUTS has a limited role and should be used selectively.

This is the first RCT with the objective of identifying differences between routine care and UDS, their hypothesis was that UDS would reduce surgery rates, but such a reduction was not identified, although in a qualitative analysis the same group identified that a key reason for men wanting to undergo UDS was its perceived value in providing additional insight to them and their clinicians (2). When they review the quality of the studies is important to highlight that there were differences between centers in the way of calibration, resting pressure amongst others, giving an Erroneous diagnosis of bladder outlet obstruction in 5.5% of the analyzed studies (3).

But there are questions of these studies that need answers, some of the patients worsen their symptoms despite the surgery so it is important to analyze the characteristics of this patients in order to know the utility of UDS, future research should focus on individual predictive factors influencing outcome of surgery UDS evidently remains important in some settings.
